# The Influencing Mechanism of Social and Cultural Adaptation for Chinese Migrant Children: A Longitudinal Intervention Study

**DOI:** 10.3389/fpsyg.2022.832871

**Published:** 2022-03-11

**Authors:** Bingbing Yu, Enguo Wang

**Affiliations:** ^1^Zhengzhou Preschool Education College, Zhengzhou, China; ^2^Life Education Research Center, Henan University, Kaifeng, China

**Keywords:** migrant children, psychological capital, self-esteem, social cultural adaptation, longitudinal intervention, intermediate model

## Abstract

With the increasing urbanization in China, the mental health problems of migrant children have attracted widespread attention. From the perspective of social cognition, psychological capital, self-esteem, and other factors are closely related to the social and cultural adaptation of the group. In order to explore the relationship among the psychological capital, self-esteem and socio-cultural adaptations of migrant children, a total of 245 Chinese migrant children were investigated with the psychological capital scale, self-esteem scale, and socio-cultural adaptation scale. At the same time, 24 months group longitudinal intervention exercises was designed scientifically, the participants were provided with it continuously to enhance their psychological capital. According to the results, there were significant differences in t-test in experiment group and control group of migrant children. At the same time, there was a significant linear correlation between predictive variables’ psychological capital and self-esteem and outcome variable’s socio-cultural adaptation. In the next step of mediating effect analysis, psychological capital and self-esteem were adopted for predicting socio-cultural adaptation. The psychological capital could not only directly affect the social and cultural adaptation of migrant children, but also indirectly affect the degree of social and cultural adaptation of migrant children through changing self-esteem. The results showed that the self-esteem mediation model fitted well with the data. Furthermore, the mediation effect accounted for 17.3% in the direct effect. Longitudinal group intervention could improve the psychological capital and social and cultural adaptation of migrant children. In a word, the research was helpful to explore the influence mechanism of migrant children’s psychological capital on social and cultural adaptation, and had certain practical value in preventing and reducing the crisis caused by the social and cultural adaptation of migrant children.

## Introduction

With the increasing urbanization in China, more and more migrant children move into cities with their families, starting a long and difficult process of social and cultural adaptation. The sociocultural adaptation of these immigrant children has always been a common concern of sociologists, anthropologists and psychologists. American scholar Rudmin (2009) defined acculturation as a psychological change caused by imitating the behavior of foreign culturists in a new culture defined. He believed that this concept consisted of many changes in personal behavior, attitude and mental health in the process of cultural adaptation. Since then, there was an increasing number of the empirical and theoretical researches about cultural adaptation. It not only broke through the model of studying cultural adaptation at the group level, but also expanded the study on the cultural adaptation, including the cross-cultural.

Berry (2002), a Canadian cross-cultural psychologist, had further clarified the connotation of social and cultural adaptation by drawing on anthropological theories and methods, which based on the research on indigenous peoples and immigrants in recent years. Berry (2002) believed that a perfect concept of social and cultural adaptation should include two levels: one was social and cultural adaptation at the group level or at the cultural level, that is, the changes of economic foundation, social structure and political organization after cultural contact; the other was the cultural adaptation of the psychological or individual behavior after cultural contact. It changed values, attitudes and identities among migrant children (Berry, 2002). Furthermore, cross-cultural adaptation could be divided into two categories: psychological adaptation and social-cultural adaptation. Among them, psychological adaptation is mainly based on emotional response. Migrant children gradually integrate the cultural factors of the new environment by improving their learning and life satisfaction, including positive experience such as happiness, self-confidence, and optimism. From the perspective of social cognition, social, and cultural adaptation focuses on the level of behavior. Behavior adaptation maximizes the accommodation of the current living environment by improving social communication skills and interpersonal communication skills in the new environment. Compared with local urban children, migrant children are troubled and affected by multicultural factors such as family conditions, social resources, and population migration restrictions, and show a lower level of mental health (Ward, 2001; Pajevic et al., 2020). According to the current relevant research, the process of social and cultural adaptation includes the acceptance of the current living environment and psychological integration with the immigration culture. At present, China’s urbanization has been gradually improving, but the problem of urban integration and adaptation of migrant workers become particularly prominent. In recent years, Berry’s classification of cross-cultural social adaptation had also been recognized and adopted by more empirical studies in the study of social and cultural adaptation of migrant children (Li et al., 2020). Psychological capital was a set of psychological concepts that could be measured, developed and helped to enhance positive mental ability (Yang and Wan, 2010; Sun et al., 2020; Wu et al., 2022). With the gradual rise of positive psychology, psychological capital, as an implicit variable of social and cultural adaptation, could protect the individual psychology under certain conditions (Xu and Xiao, 2009). Some studies have shown that social and cultural adaptation was influenced by internal and external factors. Psychological capital was the core positive psychological ability that could be effectively developed and accurately measured in the process of growth and development and could help individuals tap their potentials (Xiong and Ye, 2014). According to previous studies, psychological capital was vulnerable to the dual effects of risk and protective factors. Risk factors referred to the multiple factors that affected the individual’s sense of maladjustment, such as perception of discrimination, changes in living environment, and family financial difficulties. Protective factors referred to the comprehensive factors that could help the individual to better adapt to the environment (Luthans et al., 2014). In recent years, with the development of positive psychology, more and more researchers used the resource perspective of disadvantage-protective factor-well-adapted based on Rutter’s psychological capital mechanism theory in exploring vulnerable groups (Rutter, 2000), which was conducive to discovering protective factors in the process of migrant children’s adaptation and development, so as to promote and utilize them in the process of individual education. Domestic scholar Wang and others took migrant children as the research object to explore the social adaptation level in schools. The results showed that through the cultivation of psychological heath, migrant children had positive psychological state and development potential, and could adapt to school environment well even under adverse conditions (Yan et al., 2021). Psychological capital had also been recognized by more and more researchers as an inherent characteristic or ability. At present, domestic research had found that left-behind’s psychological capital was closely related to the level of cultural adaptation. As a predictor of social and cultural adaptability (Finch et al., 2020), rich psychological capital could help individuals better adapt to the environment. The positive psychological capital was positively correlated with individual’s life satisfaction and positive emotions, and negatively correlated with individual’s negative emotions. Self-esteem was an emotional response to self-ability and value judgment. The studies showed that self-esteem affects the individual s’ way of thinking, emotional experience and behavior management in the process of children’s growth. At present, most related studies regarded self-esteem as an important factor for mental health (Li et al., 2008). The researchers found that the protective effect of self-esteem gradually strengthened as the level of stress increased. The migrant children of high self-esteem would reduce the threat assessment on stress events, and the social adaptation was significantly better. When exploring the relationship among self-esteem, school adaptation and sense of belonging of migrant children, we found that self-esteem of migrant children played a mediating role between school adaptation and sense of belonging (Peng et al., 2012).

In this study, psychological capital and self-esteem had a certain impact on the social and cultural adaptation of migrant children. However, the variables of psychological capital were not only implicit psychological variables, it had an impact on the social and cultural adaptation of migrant children through self-esteem as an intermediary variable. The study took migrant children in junior middle school as the research object, and design a series of more reasonable group counseling and training. They carried out longitudinal psychological capital intervention for 24 months in order to produce positive results. Specifically, systematic observation was carried out once every 2 months. A total of 10 observations were made before and after. At the same time, the quantitative difference analysis was carried out to explore the influence mechanism of social and cultural adaptation of migrant children according to the results measured before and after the intervention. Moreover, the quantitative difference analysis was carried out to explore the influence mechanism of social and cultural adaptation of migrant children, and the cultivation methods of improving the negative psychology and positive psychological quality of migrant children. Two hypotheses were put forward: there was a dynamic structural model among the psychological capital, self-esteem, and social and cultural adaptation of migrant children. Longitudinal group activity intervention had a positive effect in improving migrant children’s psychological capital and social and cultural adaptation.

## Materials and Methods

### Participants

Based on easy sampling method, 273 migrant children of grade one in 10 classes from 4 schools in Henan Province of China were selected to participate in the questionnaire survey, including 3 public junior middle schools and 1 school for migrant children. In the second-grade survey, 28 migrant children were lost of contact, with a loss rate of 10.2%. The main reasons were the transfer of students dropping out of school and giving up the training of group activities. The two questionnaires were coded. 245 migrant children who provided valid questionnaires were tested before and after the two surveys. Among them, 135 (64.2%) were migrant children in public schools and 110 (35.8%) were migrant children in schools for migrant children. The average age of migrant children was 14.18 ± 1.51, and the ratio of male to female was 1:1. All participants volunteered to participate in this experiment and their physical and mental status was good. At the same time, 245 matched participants in the control group were randomly selected, and the basic conditions were the same as the previous subjects. The pre-test and post-test experimental design of the experimental group control group was designed.

### Research Tools

#### Psychological Capital Scale

Zhang et al. (2010) compiled the Positive Psychological Capital Questionnaire. The questionnaire could be divided into 26 items in four dimensions: self-efficacy, resilience, hope and optimism. Likert grade 7 was used to score all items. The score reflects the change of individual psychological capital scale.

#### Self-Esteem Scale

Self-Esteem Scale (SES) was compiled by Rosenberg (1965) to assess self-worth and general self-acceptance. The SES consists of five positive scores and five reverse scores of 10 items. Scores were divided into four grades: 1 is very consistent, 2 is consistent, 3 is inconsistent, and 4 means very inconsistent. The total score ranged from 10 to 40. The score reflects the change of individual self-esteem scale (Li et al., 2008).

#### Social and Cultural Adaptation Scale

Fan et al. (2012) revised Ward and Kennedy’s Social and Cultural Adaptation Scale. 27 projects were involved. Individual projects had been amended appropriately, such as replacing the understanding difference between Beijing and their hometown with that between their present residence and their hometown. Five points of scoring, 1 means very easy and 5 means very difficult. The score reflects the change of individual social and cultural adaptation (Fan et al., 2016).

### Procedures

#### Group Intervention Program

The study involves a longitudinal intervention. Before the study began, a series of longitudinal intervention empirical studies were conducted on migrant children. With clear, feasible, challenging and specific operational sub-goals set, parents and head teachers are invited. Activities are scheduled from April 2016 to April 2018 (except for winter and summer vacation), two lessons for 50 min every 2 months (the fourth Saturday). In the 2-year longitudinal intervention, we have adopted a series of observation and monitoring, 10 times in total. Two major monitoring points are arranged in April 2016 and April 2018, respectively, and the remaining monitoring is conducted every 2 months. The specific time arrangement is as follows: June 2016, September 2016, November 2016, February 2017, June 2017, September 2017, November 2017, and February 2018. In the process of the study, we used SPSS18.0 social science statistical software to collect the data and observed the turning point of the data. In April 2016, we conducted the first monitoring on the subjects of migrant children, that is, the pre-test. The average score of social and cultural adaptation of all subjects was 4.72. In the follow-up, except winter and summer vacation, we conducted systematic monitoring every 2 months. The average change values of scores are as follows: 4.81, 4.92, 4.96, 5.12, 5.14, 5.15, 5.21, and 5.23. During the last observation and monitoring, there was a statistically significant difference in the data, that is, 5.27, which is the data reflected in the table. Longitudinal observation of the whole data found that after a series of interventions, the data of social and cultural adaptation of migrant children gradually increased, indicating that the effect of longitudinal intervention is obvious. See Table 1 for specific series data. Organized and implemented by graduate students and school mental health counseling teachers. All researchers have received standardized training. Eight group training activities were arranged, each with a theme goal. The specific modules are arranged in a gradual and progressive order. The details are as follows: (1) Self-acceptance; (2) Development of optimism; (3) Development of self-efficacy; (4) Development of resilience; (5) Development of potential; (6) Cultivation of responsibility; (7) Learning to be grateful (8) Facing the future. Through games, role-playing, brainstorming, behavioral training, personal reflection, story rewriting, video watching, mutual evaluation and planning, the activities achieve the overall goal of enhancing the mental capital of migrant children and promoting their social and cultural adaptation. After the subjects completed the longitudinal intervention group auxiliary activities, the standardized trained researchers conducted a questionnaire survey, and then collected effective data for follow-up research. In the process of data collection, the consistency of data collection before and after the test is ensured through a unified research process.

**TABLE 1:** Descriptive statistical analysis of longitudinal series of interventions.

	2	3	4	5	6	7	8	9
Self-esteem	4.43 + 0.69	4.43 + 0.65	4.43 + 0.67	4.45 + 0.69	4.46 + 0.68	4.56 + 0.68	4.67 + 0.68	4.73 + 0.68
Psychological capital	4.46 + 1.63	4.47 + 1.72	4.67 + 1.73	4.67 + 1.75	4.67 + 1.76	4.67 + 1.78	4.67 + 1.79	5.72 + 1.65
Total score of social and cultural adaptation	5.05 + 1.29	5.16 + 1.34	5.25 + 1.36	5.35 + 1.37	5.45 + 1.38	5.53 + 1.39	5.65 + 1.38	5.78 + 1.69

#### Measurement Procedure and Data Processing

Both questionnaire surveys were conducted according to schedule. In the 2-year longitudinal intervention, we have adopted a series of observation and monitoring, 10 times in total. The first one was conducted in a collective way in the whole class. The surveyors explain the purpose, content and filling requirements of the survey to the respondents. Two major monitoring points are arranged in April 2016 and April 2018, respectively, and the remaining monitoring is conducted every 2 months. The specific time arrangement is as follows: June 2016, September 2016, November 2016, February 2017, June 2017, September 2017, November 2017, and February 2018. In the process of the study, we used SPSS18.0 social science statistical software to collect the data and observed the turning point of the data. Each monitoring is conducted by the same group of professionally trained researchers. The researchers were trained in a standardized way and kept consistent during the pre-test and post-test study. After the questionnaires being collected, migrant children were screened out and their parents, head teachers and migrant children themselves agreed to participate. After training topics are determined and commitment agreement was signed, eight group activities were conducted within 1 year, the selected migrant children were coded and then trained for 24 months. At the end of the experiment, the valid questionnaires before and after the tests were taken as the participants. The subsequent analysis used the data from both time points. The SPSS18.0 was used to analyze and process the data. The descriptive statistics, Pearson correlation, regression analysis, mediation effect analysis and structural fitting model were used to analyze the data.

## Results

### Comparison of Pre-intervention and Post-intervention Results of Social and Cultural Adaptation, Psychological Capital, and Self-Esteem Scores of Migrant Children

In order to better explore the effect of vertical group intervention to enhance psychological capital, the study first conducted a questionnaire survey on social and cultural adaptation, psychological capital and self-esteem of migrant children before intervention, and used it as a benchmark score for comparison. In the research process, we used the pre-test and post test experimental design of the experimental group and the control group to avoid the interference of the participants’ maturity factors. After a series of 24-month longitudinal intervention training activities, migrant children were tested afterward. The valid questionnaires provided by both before and after the tests were the subjects. The difference between the Experimental group and the control group was tested by *t*-test. The results showed that the self-esteem score of migrant children after intervention was slightly higher than that before intervention, and there was no significant difference. The psychological capital and social status of migrant children were not significantly different after intervention. The scores of cultural adaptation of experience group were significantly higher than those of control group before intervention (*P* < 0.01). The group activities could positively enhance the mental health and psychological capital of migrant children. The results were shown in Tables 1, 2.

**TABLE 2:** *T*-test for the difference between pre-test and post-test in Experiment group and Control group of migrant children.

	Control group		Experience group		*T*
	Preintervention	Post intervention	Preintervention	Post intervention	
Self-esteem	4.42 + 0.68	4.48 ± 0.63	4.43 + 0.69	4.73 + 0.68	0.231
Psychological capital	4.67 + 1.71	4.53 ± 1.63	4.68 + 1.72	5.62 + 1.65	−0.563**
Total score of social and cultural adaptation	5.05 + 1.39	5.43 ± 1.21	5.06 + 1.41	5.48 + 1.62	−0.214**

**P < 0.01.

### Relevance Analysis on Social and Cultural Adaptation, Psychological Capital, and Self-Esteem of Migrant Children

According to the analysis of Pearson, a British scholar, the psychological capital of migrant children was positively correlated with self-esteem and social and cultural adaptation, and self-esteem was positively correlated with psychological capital and social and cultural adaptation (*P* < 0.01). The above related studies showed that there was a significant linear correlation between predictive variables’ psychological capital and self-esteem and outcome variable’s socio-cultural adaptation. The above results pave the way for the next step of regression analysis. In the next step of mediating effect analysis, psychological capital and self-esteem were adopted for predicting socio-cultural adaptation. The results were shown in Table 3.

**TABLE 3:** Relevance analysis of social and cultural adaptation, psychological capital and self-esteem of migrant Children.

	Social and cultural adaptation	Psychological capital	Self-esteem
Self-esteem	0.018	0.150**	0.273**
Psychological capital	0.831**	0.734**	0.472**
Total score of social and cultural adaptation	0.247**	0.483**	0.215**

**P < 0.01.

### Intermediate Analysis of Self-Esteem of Migrant Children Between Psychological Capital and Socio-Cultural Adaptation

**FIGURE 1:**
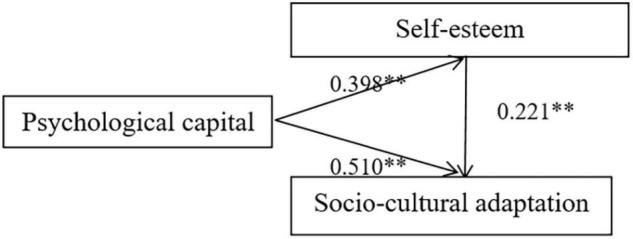
Intermediate effect of self-esteem between psychological capital and social and cultural adaptation. The values in the figure are standardized path coefficients. ^**^P < 0.01.

According to the theoretical hypothesis, the manuscript built the mediating effect model of self-esteem of migrant children in psychological capital and social and cultural adaptation. The manuscript adopted the method taken by Wen Zhonglin, a Chinese scholar, to test the procedure in turn (Wen et al., 2016). Take social and cultural adaptation score as dependent variable, psychological capital score as independent variable and self-esteem as mediating variable. The first step was the regression analysis on psychological capital to social and cultural adaptation, and the path coefficient C was obtained; the second step was the regression analysis of psychological capital to self-esteem, and the third step was to include the self-esteem and generate psychological capital. Based on the regression analysis of Ben and self-esteem, path coefficients B and C were obtained. The result shows that psychological capital positively predicted social and cultural adaptation in the first step of regression analysis; in the second step, psychological capital played a positive role in predicting self-esteem; in the third step, the intermediary variable was included in the regression equation. On the basis of controlling the influence of self-esteem on social and cultural adaptation, psychological capital still played a positive role in predicting social and cultural adaptation, and the absolute value of regression coefficient became smaller, which indicated that self-esteem started to be fostered. Rational capital played a part of intermediary role between social and cultural adaptation. The results were shown in Table 4. According to the hypothesis, we established a mediating effect map of self-esteem between psychological capital and social cultural adaptation (see Figure 1). The results showed that the path coefficient between psychological capital and socio-culture adaptation was significant (β = 0.510, *P* < 0.01), and has a significant prediction effect. The path coefficient of psychological capital on self-esteem is significant (β = 0.398, *P* < 0.01), the path coefficient of self-esteem on social and cultural adaptation was significant (β = 0.221, *P* < 0.01). The percentage Bootstrap method with deviation correction was used to test the mediation effect. The selected self-sampling volume of Bootstrap was 439. The results showed that self-esteem partially mediated the effect of psychological capital on social and cultural adaptation of migrant children. The 95% confidence interval was [0.03, 0.37], excluding 0. The results were displayed in Figure 1.

**TABLE 4:** Intermediate analysis of self-esteem of migrant children between psychological capital and socio-cultural adaptation.

	Prediction variable	Dependent variable	Modified R2	*F*	β	*T*
Path C	Psychological capital	Socio-cultural adaptation	0.258	165.417**	0.510**	12.861**
Path A	Psychological capital	Self-esteem	0.158	84.001**	0.398**	9.165**
Path B	Psychological capital	Psychological capital	0.308	99.285**	0.221**	5.154**
Path C	Self-esteem				0.429**	9.983**

**P < 0.01.

### Fitting Analysis of Intermediate Model Between Self-Esteem of Migrant Children and Psychological Capital and Socio-Cultural Adaptation

Migrant children were taken as independent variables, social, and cultural adaptation level as dependent variables, and self-esteem level as intermediary variable for analysis. Through data analysis, we can better mine the value of data and provide good guidance for research. Therefore, a structural model of the relationship between them was established. The data of the model showed that the self-esteem of migrant children plays a part of mediating role in psychological capital and socio-cultural adaptation. Together with the analysis on the mediating effect in Table 4, the proportion of mediating effect and direct effect of self-esteem level was P = b*c/(1-b*c) = 17.3%, respectively. From the various indicators provided by the model, we could see that the intermediary model of self-esteem level of migrant children fits the data well. The results were shown in Table 5.

**TABLE 5:** Fitting analysis of intermediate model between self-esteem of migrant children and psychological capital and socio-cultural adaptation.

Model	NFI	CFI 2	2/df	GFI	RMSEA
0.901	0.940	38.175	3.180	0.966	0.085

## Discussion

This study found that migrant children’s post-test psychological capital and socio-cultural adaptability were significantly improved compared with the pre-test of migrant children, which indicated that the longitudinal group intervention had a certain positive effect on improving migrant children’s psychological capital and socio-cultural adaptability after 24 months of vertical group intervention. It was consistent with the results of the group intervention activities for adolescents by domestic scholars, which could not only improve the mental health level of adolescents, but also effectively improve the psychological capital of adolescents (Fang, 2012). Through individual interviews with migrant children, their ability also improved to cope with the living environment, and there were corresponding means to adjust the pressure in the process of cultural adaptation; in the process of adjustment, they learned practical and adaptive skills and methods, and gradually accepted the rules of diet, climate, residence and society. Psychological and physiological changes, such as interpersonal communication and new cultural values, indicate that psychological capital, had a positive impact on individual emotional adjustment, learning and daily behavior. The Chinese scholars believed that the intervention on migrant children’s psychological capital would help to improve their psychological capital. However, in the process of intervention, we needed to consider the long-term effects. Only through a long-term follow-up study of intervention, we could constantly consolidate the intervention results at each stage and eventually form a strong inner world. This was consistent with the original intention and results of this study, and provided a reference for follow-up researches (Shen, 2009).

According to the research, the self-esteem of migrant children in the post-test was slightly higher than that in the pre-test, which indicated that the mental health of migrant children tends to develop slowly in the process of adapting to the new social culture, which might be related to the 24-month group counseling training for migrant children during the research. In recent years, the government had promulgated policies and measures conducive to the employment, residence and enrollment of migrant children, which reduced discrimination and rejection of migrant children by society. The migrant children could gradually integrate into the city in a better way. The improvement of the real social environment was the external force of self-esteem, which was conducive to the self-esteem of migrant children. At the same time, compared with the left-behind children in the countryside, better education, cultural resources and better material living conditions in the city provide certain support for their development and enable them to have a strong sense of self-esteem (Fan et al., 2016). The improvement of living environment had a certain impact on the self-esteem development of migrant children and the above variables.

However, compared with previous studies, it was noteworthy that although migrant children received intervention training of a series of group activities from the first to the second stage of junior high school, the development of their self-esteem level only showed a slightly upward trend, and the change was not significant. Whether the result was related to the development trend of adolescent self-esteem in the form of “U” found by domestic scholar Zhang. It was believed that in the first stage of junior middle school, the self-esteem was higher. In the second stage, due to the pressure brought by academic and interpersonal problems, adolescents’ sense of self-worth showed a downward trend. In the third stage, adolescents’ self-esteem level was relatively high. Zhang mentioned that adolescents’ self-esteem level presented the “U” shape. The above theoretical viewpoints provided evidence support for this study (Zhang, 2007). According to the law of individual development, the development of self-esteem of migrant children in adolescent groups was in the second critical period. At this stage, the group paid special attention to self-feeling and experience. In addition, the level of self-esteem was affected to a certain extent in the mobile social scenario, which would affect the overall development speed and the direction of self-esteem (Zeng, 2010).

According to the results of correlation analysis, in both the pre-test and post-test, there was a significant positive correlation between psychological capital and self-esteem and social adaptability of migrant children, and a significant negative correlation between psychological capital and self-esteem; there was a significant positive correlation between psychological capital and self-esteem; the studies showed that self-esteem played a protective role for migrant children, but most researchers regarded self-esteem as stress condition and adaptation. The regulation between outcomes was assumed to be a mediating variable. The difference lied in whether self-esteem as an individual’s internal resource could be perceived and play a role, or whether it needed to be transformed by other variables. According to Richardson’s self-esteem process model, self-esteem was regarded as the overall dynamic presentation of protective factors (Richardson, 2002). In this sense, it was more in line with the hypothesis of this study that the factors played a transformational role. The mediating effect analysis of this study also showed that the impact of self-esteem on social adaptation of migrant children was realized through psychological capital (Zhu et al., 2013).

In order to better explore the interaction mechanism among self-esteem, psychological capital and social and cultural adaptation, the path analysis method was used to explore how psychological capital affected the social and cultural adaptability of migrant children. In the research process, it was worth noting that the path analysis coefficient was different. With the self-esteem influence factor, the path coefficient increases from 0.398 to 0.429. Moreover, the study also found that self-esteem mediates the relationship between psychological capital and social adaptation of migrant children. Through structural equation model analysis, the proportion of intermediary effect and direct effect of self-esteem was 17.3%, which showed that self-esteem, as an internal psychological resource, had a certain impact on individual’s social adaptation. In this study, social and cultural adaptation was adopted as the main research object. The results showed that with more abundant psychological capital, the self-esteem of migrant children could be improved, and there was a certain regulatory effect on individual social adaptation, so that they could better adapt to the current social and cultural environment. This was consistent with many previous studies. In addition, psychological capital was featured by psychological flexibility, so the promotion of psychological capital could help individuals mobilize more social and cognitive resources, adapt to the changing living and learning environment, and improved their social and cultural adaptation.

There were also some limitations in this study. Firstly, only 2 years of intervention was conducted on the social and cultural adaptation of mechanism of migrant children, and data were collected only twice. The data showed that psychological capital not only directly affected the social and cultural adaptation of migrant children, but also indirectly affected the social and cultural adaptation of migrant children through self-esteem. However, the two data were insufficient to examine the social and cultural adaptation of migrant children and the correlation and more complex trend of change. Secondly, up to now, the relevant research mainly focused on the impact of psychological capital on the consequence variables (Xiong and Ye, 2016). Through psychological capital, migrant children’s culture was affected, but the consequence variables might also produce psychological capital in turn. The impact, such as the social and cultural adaptation level of migrant children, might also affect their psychological capital level in turn, and needed further verification in the follow-up studies. Thirdly, this study had conducted a practical study on the intervention of developing psychological capital for migrant children. In the follow-up studies, experimental studies could be carried out for different groups, such as the intervention of left-behind children’s psychological capital, so as to explore the process correlation and its effect. On the other hand, the intervention process of psychological capital would be affected by other psychological phenomena of individuals, as well as other psychological phenomena of individuals, such as the relationship between psychological capital and family environment, personality characteristics, stress events, coping styles, social support, academic performance. All these can be the focus in the next research.

## Conclusion

The psychological capital can not only directly affect the social and cultural adaptation of migrant children, but also indirectly affect the social and cultural adaptation of migrant children through the self-esteem. The longitudinal group activity intervention can significantly improve migrant children’s psychological capital and social and cultural adaptation. At the same time, it had certain practical value in preventing and reducing the crisis caused by the social and cultural adaptation of migrant children.

## Data Availability Statement

The original contributions presented in the study are included in the article/supplementary material, further inquiries can be directed to the corresponding author.

## Ethics Statement

The studies involving human participants were reviewed and approved by the Life Education Research Center of Henan University. Written informed consent to participate in this study was provided by the participants’ legal guardian/next of kin.

## Author Contributions

BY contributed to conception and design of the study, organized the database, and wrote the first draft of the manuscript. EW performed the statistical analysis and wrote sections of the manuscript. Both authors contributed to manuscript revision, read, and approved the submitted version.

## Conflict of Interest

The authors declare that the research was conducted in the absence of any commercial or financial relationships that could be construed as a potential conflict of interest.

## Publisher’s Note

All claims expressed in this article are solely those of the authors and do not necessarily represent those of their affiliated organizations, or those of the publisher, the editors and the reviewers. Any product that may be evaluated in this article, or claim that may be made by its manufacturer, is not guaranteed or endorsed by the publisher.
